# Effects of a gluten challenge in patients with irritable bowel syndrome: a randomized single-blind controlled clinical trial

**DOI:** 10.1038/s41598-022-09055-6

**Published:** 2022-03-23

**Authors:** Saeede Saadati, Amir Sadeghi, Hamid Mohaghegh-Shalmani, Mohammad Rostami-Nejad, Luca Elli, Hamid Asadzadeh-Aghdaei, Luis Rodrigo, Mohammad Reza Zali

**Affiliations:** 1 Basic and Molecular Epidemiology of Gastrointestinal Disorders Research Center, Research Institute for Gastroenterology and Liver Diseases, Shahid Beheshti University of Medical Sciences, Tehran, Iran; 2 Gastroenterology and Liver Diseases Research Center, Research Institute for Gastroenterology and Liver Diseases, Shahid Beheshti University of Medical Sciences, Tehran, Iran; 3 Center for Prevention and Diagnosis of Celiac Disease, Fondazione IRCCS Ca’ Granda Ospedale Maggiore Policlinico, Milan, Italy; 4 Gastroenterology and Liver Service, Hospital Universitario Central de Asturias, School of Medicine, University of Oviedo, Oviedo, Spain

**Keywords:** Gastroenterology, Gastrointestinal diseases

## Abstract

Non-celiac gluten sensitivity (NCGS) and irritable bowel syndrome (IBS) frequently overlap. Although, gluten-free diet (GFD) and low fermentable oligosaccharides, disaccharides, monosaccharides and polyols (FODMAP) improve the IBS clinical picture, many aspects remain unclear. Therefore, we designed a study to evaluate gluten tolerance, anxiety and quality of life in a specific study population. Fifty IBS patients were asked to follow a low FODMAP strict GFD for 6 weeks and were then randomly allocated to the following groups for a further 6 weeks: (A) receiving 8 g/day of gluten for 2 weeks; gluten-tolerating subjects received 16 g/day for 2 weeks and then 32 g/day for a further 2 weeks; (B) continuing to follow a low FODMAP strict GFD; and (C) receiving a gluten-containing diet. After the first 6 weeks, symptom scores significantly improved. Pain severity, bloating and total score were significantly decreased in the GFD and in the high-gluten groups, while the satiety score significantly increased in group C. Between-group analysis revealed significant differences for pain severity (*p* = 0.02), pain frequency (*p* = 0.04) and impact on community function (*p* = 0.02) at the end of the study. Our findings suggest that low FODMAP strict GFD could be prescribed in IBS patients and would reduce anxiety and improve the quality of life.

## Introduction

Irritable bowel syndrome (IBS), a functional lower gastrointestinal (GI) disorder, can be constant or remitting^1^. IBS is characterized by abdominal pain, bloating and irregular bowel movements^2–4^ and affects 4.1% of the population worldwide^5^, especially Asian populations (35.5% in Iran)^1^. The majority of patients experience poor quality of life and symptoms attributed to depression, anxiety and work-associated stress^3,6^. The pathogenesis of IBS is multifactorial, with food intolerance postulated for more than 30 years as a major factor triggering IBS symptoms^7,8^. Research demonstrated that long-lasting low-grade inflammation has a key role in the development of IBS^9–11^.

The etiology of IBS is largely unknown. The signs and symptoms of IBS overlap with those of different gastrointestinal disorders, such as food allergies, non-celiac gluten sensitivity (NCGS) and celiac disease^12^. NCGS is characterized by IBS-like symptoms and extra-intestinal manifestations which occur after gluten ingestion, improve rapidly after gluten withdrawal, and relapse quickly again after gluten challenge^13^. Growing evidence suggests that IBS and NCGS overlap due to their similar presentations^14–16^. In this context, the Salerno Experts suggested a gluten challenge as the reference standard to confirm NCGS^2^.

NCGS may induce IBS-like symptoms, but without the characteristics of malabsorption as in “classic” celiac disease. It is still debated whether these two entities are linked disorders or distinct diseases. In any case, exposure to food antigens in a genetically susceptible subjects may produce an abnormal immune responses^15^. At present, by definition, NCGS and IBS are two separate diseases; however, understanding their pathogenesis may help to characterize the main triggers fueling gluten sensitivity and post-infectious IBS^12^.

Several studies provided valuable insights into the etiology of symptoms and suggested that that many IBS individuals may be intolerant to different food^17^ as gluten, lactose, milk protein, and fermentable oligo-di-monosaccharides and polyols (FODMAP)^2^. A randomized clinical trial showed that removal of gluten from the diet ameliorated IBS symptoms^18^, although a subsequent trial by the same authors reported different conclusions^19^. Furthermore, there is evidence that combining FODMAPs with a gluten-free diet (GFD) substantially improves the quality of life of IBS patients^20^.

A major *dilemma* in medical practice is the amount of gluten that IBS patients can tolerate and the effect of different doses of gluten on symptomatic onset. For this purpose, we designed a single-blind, controlled gluten challenge to evaluate gluten tolerance, anxiety and quality of life among IBS patients. In addition, the prevalence of NCGS was reported.

## Materials and methods

### Participants

Eligible patients with IBS were recruited between April 2017 and August 2019 from an outpatient gastroenterology clinic in Taleghani Hospital, Tehran, Iran. The inclusion criteria were adults aged 18–80 years old fulfilling symptoms of IBS according to the ROME-IV consensus including recurrent abdominal pain on average at least 1 day/week over the last 3 months, associated with two or more of the following criteria: (1) changes in defecation, (2) changes in frequency, and (3) changes in form of stool, with no medication to alleviate symptoms in the previous 3 months^21^. Exclusion criteria were: being on a GFD or low-FODMAP diet in the previous 6 months, psychological disorders, changing the diet during the study, lack of desire to continue, major abdominal surgery, diabetes mellitus and pregnancy. Anti-Tissue Transglutaminase (Anti-tTG) and/or endomysial antibodies (EMA), histological findings compatible with atrophy (according to the Marsh classification) and wheat-specific Immunoglobulin E (IgE) levels were negative in all participants.

### Study design and intervention

**Figure 1:**
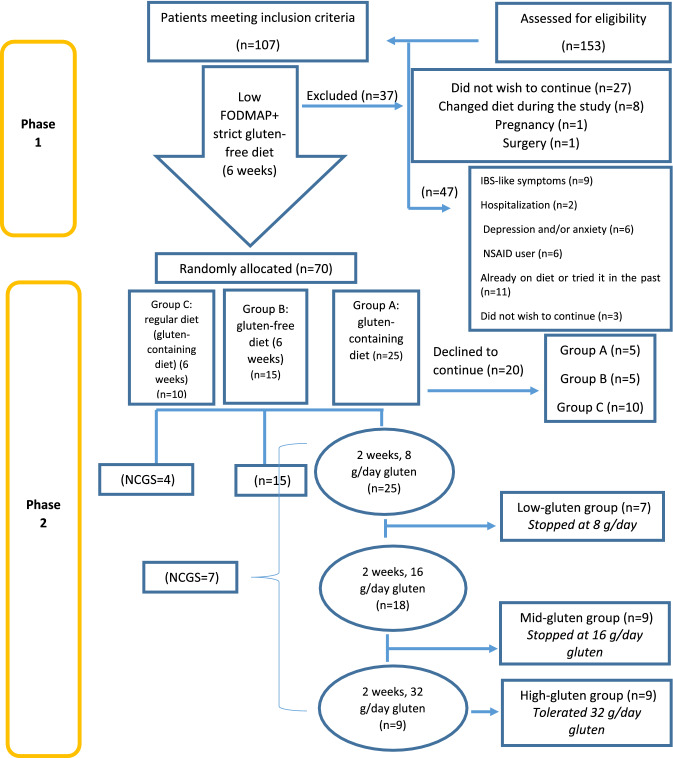
Study flowchart.

The design of this randomized clinical trial is shown in Fig. 1. Among 153 patients attending the IBS clinic, 107 consecutive newly diagnosed IBS patients who met the inclusion criteria were recruited. Forty-seven patients were excluded because of inflammatory bowel disease or other gastrointestinal comorbidities (n = 9), history of hospitalization (n = 2), depression and/or anxiety (n = 6), non-steroidal anti-inflammatory drugs (NSAIDs) users (n = 6), patients already on a therapeutic diet or who had tried one in the past months (n = 11), and those unwilling to continue the trial (n = 3). The study was conducted in two phases.

#### Phase 1

All participants were informed about the study protocol and signed an informed consent form. The demographic characteristics and medical background of all patients including food intolerances and comorbidities were recorded. Participants were asked to fill in a questionnaire about their anxiety levels (Zung Self-Rating Anxiety Scale, SAS) and their mental and physical health (36-Item Short Form (SF-36) Health Survey) and mark a visual analogue scale (VAS) for symptoms.

The SAS is a 20-item rating instrument for anxiety disorders. A score of 20–44 indicates normal anxiety levels, 45–59 mild to moderate anxiety levels, 60–74 marked to severe anxiety levels, and 75 and above extreme anxiety levels^22^.

Quality of life was evaluated using a Persian version of the SF-36 which has been validated across an Iranian population^23,24^.

A symptom questionnaire with an array of 10-cm VASs was used to assess satisfaction with the participant’s health condition and the severity of specific symptoms (bloating, abdominal pain, satisfaction with stool consistency, number of daily defecations, and overall symptoms) weekly. A score of 0 indicated no symptoms, while 10 represented extremely severe symptoms^2^.

After initial evaluation, all 107 participants were asked to follow a low-FODMAP strict GFD for 6 weeks. The dietary intervention was delivered by an experienced dietician based on the National Institute for Health and Care Excellence (NICE) guidelines^25^. Compliance with the diet was investigated through recording dietary recall for 3 days (one weekend and two work days). Energy and nutrient data were collected before and after the intervention. Dietary intake was evaluated using Nutritionist V software (First Databank, Hearst Corporation, San Bruno, CA, USA). All participants were in contact with the dietician by email and/or telephone. The value of every symptom was recorded weekly. At the end of phase 1, participants were again asked to mark VASs to evaluate improvement in symptoms. After the end of this phase, a qualified nutritionist designed an unrestricted diet to provide daily nutritional requirements, at which time phase 2 started.

#### Phase 2

Thirty-seven of the original 107 patients dropped out (Fig. 1) meaning 70 patients completed phase 1. However, 20 (five in groups A and B, and 10 in group C) declined further participation; thus, 50 patients were randomly allocated to one of three further groups.

Group A (gluten-containing diet) received a special bread (cooked, homogeneously distributed gluten) containing 8 g/day of vital gluten (low-gluten diet) for the first 2 weeks (25 patients). Those who did not experience at least 30% worsening of their symptoms according to VAS scores after the 2-week low-gluten diet continued the gluten challenge with a special bread containing 16 g/day vital gluten (mid-gluten diet) for the next 2 weeks. The level of gluten was then increased again to 32 g/day (high-gluten diet) for a third 2-week period for those patients tolerant to the special bread containing 16 g/day gluten. The patients who completed the gluten challenge with 32 g/day gluten could then undertake a completely unrestricted diet for 2 more weeks. Those who did not tolerate the 8 g or 16 g phase were considered “non-responders” and discontinued the study.

Group B continued the low-FODMAP GFD for the next 6 weeks (15 patients; despite adding blind gluten free foods to their diet).

Group C followed a completely unrestricted diet (gluten-containing diet) for next 6 weeks (10 patients). Those who experienced symptoms followed a low-FODMAP GFD to determine if they had NCGS.

This was a dietary trial and the dietitian was not blinded. However, patients in groups A and B were blinded during the phase 2 as they did not know whether their diet is gluten included or not. Patients who did not continue the challenge were excluded from further evaluation.

According to the Salerno Experts’ Criteria, subjects with a reduction of at least 30% in one of their reported symptoms, for at least 50% of the observation time, after 6 weeks of a low-FODMAP strict GFD in group A or C were categorized as NCGS. At the end of phase 2, participants were asked to mark VASs to evaluate the improvement in symptoms, and to fill in the SF-36 and SAS questionnaires^2^. The primary end-point was the severity of symptoms (evaluated by VASs), while the secondary end-points were anxiety (evaluated by the SAS) and level of mental and physical health (evaluated by the SF-36).

### Preparation of low FODMAP-GFD and special bread

GFD was obtained by allowing the consumption of GF cereals or pseudo-cereals, such as rice, buckwheat, corn, millet and quinoa (all naturally gluten-free foods with low FODMAP content). Additionally, only the consumption of vegetables and fruit poor in FODMAP was allowed. The recommended vegetables were green beans, fennel, carrots, zucchini, pumpkin, cucumbers, celery, tomato, lettuce, fennel, olive, cucumber, pumpkin, pepper and radishes, while fruits permitted were banana, blueberry, strawberry, raspberry, melon, white melon, pineapple, citrus fruits and kiwi^26^.

Gluten is a sticky, strong and elastic protein. The addition of water to flour causes hydration of the gliadin and glutenin proteins and leads to the formation of gluten in the wheat flour. In this study, the ‘special bread’ was made by mixing water, yeast and Vital Wheat Gluten flour (Bob’s Red Mill, Milwaukie, OR). Gluten concentration was determined using sodium dodecyl sulfate polyacrylamide gel electrophoresis^27,28^ and the Kjeldahl method (BÜCHI K-350, Flawil, Switzerland)^29^. Flour protein made up 66% of the total flour content and all protein was gluten (8 g dose = 12 g flour, 16 g dose = 24 g flour, and 32 g dose = 48 g flour).

### Ethics and approvals

This study was reviewed and approved by the ethics committee of the Gastroenterology and Liver Diseases Research Center, Research Institute for Gastroenterology and Liver Diseases, Shahid Beheshti University of Medical Sciences, Tehran, Iran (IR.SBMU.RIGLD.REC.1396.154) and conformed with CONSORT 2010 guidelines^30^. This study was conducted in accordance with the guidelines established in the Declaration of Helsinki^31^. It was also registered in the Iranian Registry of Clinical Trials on 20/10/2018 with the clinical trial registration number of IRCT20100524004010N26.

### Statistical analysis

Data were expressed as the mean ± standard deviation for numeric data and frequency (percent) for categorical data and compared with t-test. Data were compared between the five groups (low-gluten, mid-gluten, high-gluten, GFD and unrestricted gluten-containing diet groups). Within-group comparison was also made for each group. The chi-squared test, or if required, Fisher’s exact test, was used for categorical data. Non-parametric grouped data were expressed as means (95% CI) and compared using the Mann–Whitney rank sum test (unpaired) or Wilcoxon’s signed-rank test (paired). A *p*-value less than 0.05 was considered significant. Comparisons between each two groups were analyzed by Mann–Whitney U test using Bonferroni correction.

### Informed consent statement

All study participants provided written informed consent (signed and dated).

## Results

### Recruitment

107 patients were eligible to enter the study and 70 completed phase 1. Fifty of these completed phase 2 and their data were entered in the final analysis (Fig. 1).

### Baseline data

Among the 50 patients who completed the study, 26 (52%) were male, most (35, 70%) were married and some had gone to school or university (20, 40%). Some IBS cases (21, 42%) were subcategorized as constipation-predominant IBS (IBS-C). No statistical differences were detected when the type of IBS in the five groups was compared. Table 1 reports the demographic and clinical variables of the groups. Statistical analysis showed no significant differences among the five groups regarding age, Body mass index (BMI), sex, marital status, smoking or education.

**Table 1:** Baseline characteristics of participants with irritable bowel syndrome (IBS) before intervention.

	Group A			Group B	Group C	*p*-value^†^
	Low-gluten (n = 7)	Mid-gluten (n = 9)	High-gluten (n = 9)	GFD (n = 15)	Normal diet (n = 10)	
Age (years)	43.1 ± 8.9* (35–57)	31 ± 5.6 (22–38)	37.2 ± 11.9 (21–59)	35.8 ± 10.6 (18–57)	37.5 ± 10.1 (27–55)	0.18
Weight (kg)	70.7 ± 8.12 (60–81)	73.3 ± 9.34 (63–88)	67.9 ± 11.2 (52–84)	74.7 ± 11.3 (55–91)	73.4 ± 12.8 (54–96)	0.69
BMI (kg/m^2^)	25.3 ± 2.1 (22.5–28.8)	26.8 ± 3.7 (23.6–35.3)	25.6 ± 4.8 (18.7–33.2)	26.1 ± 4.4 (20.3–37)	24 ± 4.6 (17–32.1)	0.60
**Gender**						0.81
Male	4 (57.1%)	3 (33.3%)	3 (33.3%)	8 (53.3%)	8 (80%)	
Female	3 (42.9%)	6 (66.7%)	6 (66.7%)	7 (46.7%)	2 (20%)	
**IBS type**						0.81
C	4 (57.1%)	4 (44.5%)	3 (33.3%)	5 (33.3%)	5 (50.0%)	
D	2 (28.6%)	3 (33.3%)	3 (33.3%)	7 (46.7%)	4 (40.0%)	
M	1 (14.3%)	2 (22.2%)	1 (11.1)	2 (13.3%)	1 (10.0%)	
U	0	0	2 (22.3%)	1 (6.7%)	0	
**Marital status**						0.25
Single	0	4 (44.4%)	4 (44.4%)	5 (33.3%)	2 (20.0%)	
Married	7 (100%)	5 (55.6%)	5 (55.6%)	10 (66.7%)	8 (80.0%)	
**Education**						0.74
Primary	1 (14.2%)	2 (22.2%)	3 (33.3%)	4 (26.7%)	2 (20.0%)	
Secondary	3 (42.9%)	1 (11.1%)	3 (33.3%)	6 (40.0%)	5 (50.0%)	
University	3 (42.9%)	6 (66.7%)	3 (33.4%)	5 (33.3%)	3 (30.0%)	
**Smoking**						0.18
Yes	0	2 (22.2%)	2 (22.2%)	1 (6.7%)	4 (40.0%)	
No	7 (100%)	7 (77.8%)	7 (77.8%)	14 (93.3%)	6 (60.0%)	

Group A: Patients receiving special bread containing 8 g/day gluten for 2 weeks; gluten-tolerating subjects received 16 g/day for 2 weeks and then 32 g/day for a further 2 weeks. Group B: Patients following a low-FODMAP strict GFD for 6 weeks. Group C: Patients receiving a regular gluten-containing diet for 6 weeks.

IBS type: IBS-C (constipation), IBS-D (diarrhea), IBS-M (mixed) and IBS-U (unsubtyped).

*Mean ± standard deviation (min–max).

^†^Using the Kruskal–Wallis test for numeric data and the chi-square test for categorical data.

Table 2 outlines the dietary recall information of participants who adhered strictly to the low-FODMAP strict GFD in phase 1 of the study. As shown in the table, there were no significant differences over 6 weeks regarding energy, macronutrients and micronutrients, with the exception of total fat, fiber, SFA and MUFA (*p* < 0.0.5).

**Table 2:** Energy intake and nutrient consumption of participants at baseline and after 16 weeks of study.

	Baseline (mean ± SD)	After 12 weeks (mean ± SD)	*p*-value
Energy (kcal/day)	1623.12 ± 538.18	1423.87 ± 316.11	0.07
Carbohydrates (g/day)	211.85 ± 83.11	185.49 ± 59.30	0.09
Protein (g/day)	53.29 ± 12.29	53.16 ± 28.70	0.68
Fat (g/day)	62.36 ± 22.41	49.12 ± 12.81	0.03
Fiber (g/day)	20.32 ± 11.75	16.14 ± 7.87	0.01
Cholesterol (mg/day)	201.29 ± 123	211.67 ± 136.41	0.75
SFA (g/day)	21.24 ± 9.41	17.25 ± 6.89	0.01
MUFA (g/day)	22.24 ± 11.56	16.99 ± 7.59	0.03
PUFA-w6 (g/day)	2.91 ± 3.31	4.41 ± 2.22	0.45
PUFA-w3 (g/day)	1.88 ± 0.23	1.19 ± 1.73	0.61
Vitamin E (mg/day)	11.35 ± 6.51	10.46 ± 4.19	0.68
Vitamin C (mg/day)	87.11 ± 100.02	73.91 ± 68.43	0.48
Zinc (mg/day)	7.38 ± 2.23	5.31 ± 3.45	0.27
Selenium (μg/day)	61.57 ± 32.15	53.29 ± 20.84	0.14

### VAS scores

According to Table 3, within-group analysis showed a significant decrease in pain severity (from 2.9 ± 2.8 to 2 ± 2.2, *p* = 0.02) and bloating (from 3.5 ± 2.6 to 2.5 ± 2.1, *p* = 0.03) in the GFD group, and a significant increase in the satiety score (from 2.3 ± 1 to 3.8 ± 3.1, *p* = 0.04) in the unrestricted gluten-containing diet group. Total score was significantly decreased in the high-gluten group from 45.6 ± 15.9 to 27.8 ± 12.8 (*p* = 0.05).

**Table 3:** Comparison of VAS scores within groups and between groups at the end of the study [Mann–Whitney U test was used for testing each two groups. There were no significant differences between each two groups, except between low gluten and high gluten diet for abdominal pain (p = 0.002), and mid gluten and high gluten diet for impact on community function (p = 0.003)].

Group A									Group B			Group C			*p*-value^†^
Low-gluten (n = 7)			Mid-gluten (n = 9)			High-gluten (n = 9)			GFD (n = 15)			GCD (n = 10)			
Baseline	End	*p*-value*	Baseline	End	*p*-value*	Baseline	End	*p*-value*	Baseline	End	*p*-value*	Baseline	End	*p*-value*	
Pain (severity)															
4 ± 2.9	5.4 ± 2.8	0.11	4.1 ± 3.5	3.3 ± 2.9	0.28	2.4 ± 2.1	1 ± 1.1	0.14	2.9 ± 2.8	2 ± 2.2	**0.02**	1.1 ± 1.6	1.9 ± 2.3	0.13	**0.02**
Pain (frequency)															
4.6 ± 4.2	7 ± 3.7	0.10	1.3 ± 1.5	1.3 ± 1.5	1	0.7 ± 0.5	1 ± 1.4	0.68	1.9 ± 2.4	2.3 ± 3.3	0.54	0.7 ± 1.1	2 ± 2.8	0.07	0.048
Bloating															
3.1 ± 2.9	3.6 ± 2.6	0.92	4.4 ± 1.9	4.1 ± 1.5	0.49	2.7 ± 2.1	2 ± 2.2	0.35	3.5 ± 2.6	2.5 ± 2.1	**0.03**	2.3 ± 2.8	3.5 ± 3.9	0.12	0.35
Satiety															
2.3 ± 1.9	4.1 ± 2.5	0.12	2.4 ± 1.6	2.7 ± 1.2	0.67	1.7 ± 1.7	1.9 ± 2	0.71	3.6 ± 2.2	3.1 ± 1.9	0.08	2.3 ± 1	3.8 ± 3.1	0.04	0.20
Impact on community function															
3.4 ± 2.6	4.3 ± 2.8	0.34	4.8 ± 2.1	5.1 ± 1.5	0.52	2.9 ± 2	2.1 ± 1.6	0.39	3.6 ± 2.6	2.7 ± 2	0.07	2 ± 1.6	3 ± 2.3	0.06	**0.02**
Defecation (times/day)															
1 ± 1	0.9 ± 0.7	0.71	1.3 ± 0.5	1 ± 0.5	0.26	1.6 ± 1	2 ± 1.1	0.19	1.8 ± 1.9	1.4 ± 0.7	0.74	1.3 ± 1.1	1.3 ± 0.8	1	0.87
Defecation (type)															
3.6 ± 1.6	3.4 ± 1.6	0.32	3.8 ± 1.2	4.2 ± 0.7	0.4	4 ± 0	4.4 ± 0.9	0.16	3.9 ± 0.7	3.8 ± 0.8	0.68	4.1 ± 0.7	3.9 ± 0.9	0.59	0.15
Total score															
47.1 ± 19.8	54.3 ± 16.2	0.27	51.1 ± 11.9	51.1 ± 15.6	0.75	45.6 ± 15.9	27.8 ± 12.8	**0.05**	44.3 ± 22.6	37.7 ± 18.9	0.06	38 ± 20	42.5 ± 21.8	0.29	**0.054**

*p-value for comparison within each group using the Wilcoxon-signed rank test.

^†^*p*-value for between-groups comparison using the Kruskal–Wallis test.

Among patients in group A, seven tolerated 8 g/day gluten, nine 16 g/day gluten and nine 32 g/day gluten, and eight individuals were diagnosed as NCGS. Comparison of the low-gluten, mid-gluten and high-gluten groups showed that patients on the low-gluten diet had higher scores at the end of the study, although no significant differences were found in each group. Four patients in group C were diagnosed as NCGS.

At the end of the study, patients were re-evaluated for IBS symptoms and the scores were compared between all groups. As presented in Table 3, between-group analysis revealed significant differences for pain severity (*p* = 0.02), pain frequency (*p* = 0.04) and impact on community function (*p* = 0.02) at the end of the study.

There were no significant differences between each two groups, except between low gluten and high gluten diet for abdominal pain (p = 0.002), and mid gluten and high gluten diet for impact on community function (p = 0.003).

### SF-36 questionnaire

About SF-36, patients in groups A and B reported significantly higher scores for ‘physical functioning’ (*p* < 0.05). The GFD group had a higher score for ‘role limitation due to physical health’ (5.9 ± 1.5 at baseline vs. 6.5 ± 1.5 at the end of the study, *p* = 0.02), as well as a significantly lower score for ‘energy/fatigue’ (14.9 ± 1.8 at baseline vs. 13.8 ± 1.5 at the end of the study, *p* = 0.01) and ‘bodily pain’ (6.6 ± 1.9 at baseline vs. 4.7 ± 2.3 at the end of the study, *p* = 0.002) at the end of the study. Also, a significantly decreased score for ‘bodily pain’ was observed in the unrestricted gluten-containing diet group (5.9 ± 1.8 at baseline vs. 3.3 ± 1 at the end of the study, *p* = 0.02).

At the end of the study, no significant difference was reported between the groups in terms of ‘physical functioning’, ‘role limitation due to physic health’, ‘role limitation due to emotional problems’, ‘emotional well-being’, ‘social functioning’, ‘bodily pain’ and ‘general health’. Table 4 presents the SF-36 results for the five groups, with within- and between-group comparisons. No significant differences were observed between each two groups.

**Table 4:** Mean ± SD scores for Short Form-36 (SF-36) Health Survey scales within groups and between groups at the end of the study (Mann–Whitney U test was used for testing each two groups. No significant differences were observed between each two groups).

Group A									Group B			Group C			*p*-value^†^
Low-gluten (n = 7)			Mid-gluten (n = 9)			High-gluten (n = 9)			GFD (n = 15)			Unrestricted (n = 10)			
Baseline	End	*p*-value*	Baseline	End	*p*-value*	Baseline	End	*p*-value*	Baseline	End	*p*-value*	Baseline	End	*p*-value*	
Physical functioning															
26.6 ± 4.7	28.7 ± 2	0.04	22.6 ± 1.9	25.2 ± 1.8	0.02	23.7 ± 4.7	25.9 ± 3.9	0.03	26.2 ± 3.5	27.6 ± 2.8	0.02	27.4 ± 2.6	26.1 ± 3.7	0.50	0.053
Role limitation due to physical health															
6.3 ± 1.5	7 ± 1.2	0.06	5.9 ± .9	6 ± 1	0.56	6 ± 1.6	6.1 ± 1.5	0.66	5.9 ± 1.5	6.5 ± 1.5	0.02	6.9 ± 1.5	6.1 ± 2	0.06	0.59
Role limitation due to emotional problems															
4.1 ± 1.2	5.1 ± 1.1	0.17	4.7 ± 1.4	5.1 ± 1.3	0.36	4.6 ± 1.3	4.9 ± 1.2	0.18	4.5 ± 1.5	4.9 ± 1.4	0.06	4.4 ± 1.3	4 ± 1.3	0.10	0.39
Energy/fatigue															
14.4 ± 2.5	14.7 ± 2.4	0.34	16.8 ± 3.4	16.1 ± 3.5	0.55	14.8 ± 1.1	14.3 ± 1	0.21	14.9 ± 1.8	13.8 ± 1.5	0.01	14.8 ± 2.4	13.6 ± 1.7	0.25	0.15
Emotional well-being															
22.7 ± 1.6	21.6 ± 2.5	0.46	22.2 ± 2	21.8 ± 1.9	0.38	19.6 ± 3	20.3 ± 2.1	0.27	20.7 ± 2.5	21.2 ± 1.9	0.29	19.6 ± 3.1	19.4 ± 3.5	0.83	0.31
Social functioning															
5.6 ± 1.1	5.7 ± 1.7	0.71	5.9 ± 2.4	5.11 ± 1.364	0.13	6.6 ± 1.2	5.7 ± 0.7	0.054	5.9 ± 1.5	5.8 ± 1.3	0.72	6.7 ± 1.4	6.1 ± 2	0.32	0.56
Bodily pain															
4.6 ± 2.3	3.7 ± 1	0.34	5.4 ± 2.6	4.00 ± 1.118	0.26	5.2 ± 1.7	4.4 ± 1.5	0.08	6.6 ± 1.9	4.7 ± 2.3	0.002	5.9 ± 1.8	3.3 ± 1	0.02	0.56
General health															
17.1 ± 2.3	16.1 ± 2.6	0.07	19.7 ± 2	18.00 ± 2.693	0.058	17.9 ± 2.7	17.8 ± 2.5	0.71	16.9 ± 2.7	16.2 ± 1.9	0.31	16.7 ± 2.1	15.6 ± 2.6	0.34	0.23

*p-value for comparison within each group using the Wilcoxon-signed rank test.

^†^*p*-value for between-groups comparison using the Kruskal–Wallis test.

Higher scores in the SF-36 indicate better health. Differences across groups A, B and C were analyzed with a linear mixed model and *p-*values are given for the main effect of gluten challenge. *SD* standard deviation.

### Zung self-rating anxiety scale (SAS)

The SAS showed that only one patient in the high-gluten group with mild to moderate anxiety levels (score 46) before the study remained at the same level (score 48) at the end of the study. In the GFD group, continuing the GFD significantly decreased the anxiety total score (*p* = 0.005), especially the somatic manifestations of anxiety including feeling a rapid heartbeat (*p* = 0.046), dry and warm hands (*p* = 0.046), and hot flushes (*p* = 0.008). Nervousness in the unrestricted gluten-containing diet group (*p* = 0.03), and stomach pain in each group had significantly decreased at the end of the study (*p* < 0.05). There were significant differences in anxiety total score between baseline and the end of the study in the GFD (*p* = 0.005), low-gluten (*p* = 0.03) and mid-gluten (*p* = 0.02) groups (Table 5). There were no significant differences between each two groups.

**Table 5:** Total scores on the Zung Self-Rating Anxiety Scale (SAS) in irritable bowel syndrome (IBS) patients within groups and between groups at the end of the study (Mann–Whitney U test was used for testing each two groups. There were no significant differences between each two groups).

Group A									Group B			Group C			*p*-value^†^
Low-gluten (n = 7)			Mid-gluten (n = 9)			High-gluten (n = 9)			GFD (n = 15)			Unrestricted (n = 10)			
Baseline	End	*p*-value*	Baseline	End	*p*-value*	Baseline	End	*p*-value*	Baseline	End	*p*-value*	Baseline	End	*p*-value*	
36 ± 3.2	33.7 ± 3.6	0.03	39.6 ± 3.3	35.2 ± 2.1	0.02	36.7 ± 4.8	35.7 ± 5.3	0.11	38.6 ± 4.8	32.7 ± 3.6	0.005	35.9 ± 3.7	33.6 ± 3.6	0.06	0.29

*p-value for comparison within each group using the Wilcoxon-signed rank test.

^†^*p*-value for between-groups comparison using the Kruskal–Wallis test.

## Discussion

In the present study, we evaluated gluten tolerance and its effects on the somatic and psychological manifestations of IBS. In phase 1, patients followed a strict GFD and low-FODMAP diets for 6 weeks. The mean score of all symptoms had significantly decreased at the end of phase 1. A low-FODMAP diet and GFD are currently used separately and in combination for the treatment of IBS. Clinical trials have shown the efficacy of GFD on IBS symptoms. Vazquez–Roque et al. demonstrated increased bowel movements and greater intestinal permeability in a gluten- containing diet group compared with a GFD group^27^. Additionally, among celiac patients meeting IBS criteria, adding a low-FODMAP diet to a GFD resulted in a dramatic response *vs* usual GFD. A considerable reduction in the VAS for abdominal pain and a greater improvement in general well-being were noted in the low-FODMAP and GFD combination group^28^.

In phase 2, after 6 weeks long low-FODMAP strict GFD, all patients were randomly allocated to: an unrestricted daily gluten-containing diet, continued gluten-free/low-FODMAP diets, and a gluten challenge group (low-gluten diet (8 g/day gluten), mid-gluten diet (16 g/day gluten) and high-gluten diet (32 g/day gluten)). We did not find any significant differences between the groups in terms of bloating, satiety, defecation or total score. The same results have been reported in a study by Biesiekierski et al., in which no dose-dependent effects of gluten on IBS subjects were observed. The authors randomly prescribed high-gluten (16 g/day gluten) and low-gluten (2 g/day gluten and 14 g/day whey protein) diets for a week, followed by a washout period of at least 2 weeks in 37 patients with NCGS and IBS^19^. The evidence mostly supports the effects of gluten on the symptoms of patients with IBS. These conflicting results in patients undergoing gluten challenge, further highlights the fact that the etiology of IBS is unclear. Possibly, there is a subgroup of IBS, categorized as gluten sensitivity-IBS (GS-IBS), with a different mechanism. Alternatively, GS-IBS might be a new medical term or a subclass of gluten-related disorders^32^.

Of note, we evaluated symptoms but did not assess objective outcomes. We educated patients on GFD and how avoid gluten-containing foods, including wheat, barley and rye. However, components of wheat other than gluten may be responsible for increasing symptoms in IBS patients. Indeed, we did not observe the development or aggravation of symptoms after gluten challenge in our study. A recent study has suggested that fructans, rather than gluten, are responsible for symptoms observed in patients with IBS. Skodje et al. prescribed diets containing fructans, placebo and gluten for 7 days in 59 subjects on GFD in a double-blind crossover trial. The overall gastrointestinal symptom rating scale (GSRS) score was higher in those who took fructans compared to gluten consumers^33^. Since the main trigger of NCGS is not known, a GFD is currently recommended in NCGS. However, patients with NCGS do not have to follow a GFD as strictly as patients with celiac disease. Although some studies show that patients with IBS benefit from a GFD, recent ones have shown the positive effect of a low-FODMAP diet in IBS^20^. Furthermore, within-group comparison did not show any significant difference overall; however, an increased satiety score and decreased pain severity, as well as bloating were observed in the GFD group following an unrestricted diet.

Regarding the SF-36 questionnaire, patients in the GFD group reported a significantly higher score for ‘role limitation due to physical health’, as well as a significantly lower score for ‘energy/fatigue’ and ‘bodily pain’ at the end of the study. Also, a significant decrease in the social functioning score was observed in the high-gluten group and in the bodily pain score in the unrestricted gluten-containing diet group. Patients in groups A and B reported a significantly higher score for ‘physical functioning’. These results show that those who consumed gluten during the study (unrestricted group) had worse physical functioning, reflecting the adverse physical effects of gluten consumption on IBS patients. At the end of the study, no significant difference was reported between groups in terms of the different quality of life domains (Table 4). It has been established that IBS significantly alters health-related quality of life (HR-QoL) among affected patients, and response to treatment is associated with improvement in HR-QoL^34^. IBS patients worry about what foods cause symptoms so that they can avoid eating them. This particularly applies for diets high in carbohydrates and fats and rich in biogenic amines, as well as histamine-releasing foods^35^.

A GFD is mandatory in patients with celiac disease and could have beneficial effects on signs, symptoms and reduce the risk of associated complications^36^. Nonetheless, a strict adherence to GFD is problematic^37^ and affected by difficulty in finding gluten-free foods, their altered taste and high cost, lack of food labelling and deteriorated sociality^37,38^. Following a GFD might also result in nutritional deficiencies, such as a lack of dietary fiber, vitamin D, calcium and magnesium^39,40^. Thus, these different effects of following a GFD on the personal and social aspects of affected individuals impact their quality of life. Rodrigo et al. showed the effectiveness of a 1-year GFD on improving quality of life and decreasing VAS scores among seven adult female celiac patients with severe IBS and fibromyalgia^41^. A study by Hallert et al. showed that despite following a GFD for 10 years, adult celiac patients still had a lower quality of life than the general population, especially women, who had more gastrointestinal symptoms on the GSRS compared with men^42^. Similarly, in a national survey in Germany, decreasing severity of symptoms was not associated with improvement in quality of life^43^. These data suggested that, apart from normalization of bowel mucosa and a reduction in gastrointestinal symptoms, other factors are involved in the quality of life of these patients.

Drossman has suggested a biopsychosocial model for IBS in which the interaction of biological and psychosocial factors induces the clinical expression of this disease^44^. Psychological disorders, such as anxiety and depression, are more prevalent in IBS patients than in the general population, and psychological issues are important factors in the severity and duration of symptoms as well as response to treatment^45^. Research suggests that other than toxigenic organisms, hypochondria and adverse life events increase the risk of post-infectious IBS^46^.

There was no significant difference in the anxiety total score between baseline and the end of the study in the unrestricted gluten diet group. This is compatible with a study which revealed that GFD had reduced anxiety, but depression was not affected^47^. Another German study showed that anxiety is higher in female celiac patients on a GFD^48^. Aziz et al. in their study suggested a beneficial impact of GFD in improving somatic symptoms as well as anxiety and depression in patients with IBS, diarrhea type^49^. Rostami-Nejad et al. recently reported that anxiety symptoms are common among celiac disease patients, and a GFD does not improve them^50^. We found that a GFD reduced abdominal pain and bloating in the GFD group, which may explain the improvement in anxiety in the IBS patients following a GFD.

The limitations of this study include the small number of patients and limited follow-up time. IBS is a complicated disorder with unknown etiology and its treatment requires a multi-disciplinary approach. Our study provides new research for further evaluating dietary intervention in the management of IBS.

## Conclusions

The results of this study demonstrate that most of the IBS patients in group A were tolerant to high doses of gluten. On the other hand, a low-FODMAP strict GFD had a significant effect on the anxiety in groups A and C and quality of life in groups A and B and these results led us to consider prescribing a low-FODMAP strict GFD as a therapeutic choice for the management of patients with IBS.
